# Semen parameters and male reproductive potential are not adversely affected after three or more months of recovery from COVID-19 disease

**DOI:** 10.3389/frph.2022.1114308

**Published:** 2023-01-20

**Authors:** Sara Stigliani, Claudia Massarotti, Francesca Bovis, Elena Maccarini, Paola Anserini, Paola Scaruffi

**Affiliations:** ^1^ UOS Physiopathology of Human Reproduction, IRCCS Ospedale Policlinico San Martino, Genova, Italy; ^2^ Department of Neuroscience, Rehabilitation, Ophthalmology, Genetics and Maternal-Child Health (DiNOGMI), University of Genova, Genova, Italy; ^3^ Department of Health Sciences (DISSAL), University of Genova, Genova, Italy

**Keywords:** COVID-19, semen analysis, male fertility, IVF, male reproduction

## Abstract

**Background:**

The male reproductive system may be a potential target for SARS-CoV-2 since the presence of ACE and TMPRS2 receptors. After a first report of the presence of SARS-CoV-2 in semen of COVID-19 patients, several papers reported that SARS-CoV-2 was not detected in the semen. However, some evidences indicated that COVID-19 disease could impair semen parameters. During the infection, or in a short period after, a reduction in sperm concentration and motility and an increase in DNA fragmentation were observed, even in asymptomatic patients. There is no conclusive data exploring whether this damage changes with time. We investigated whether COVID-19 disease has a negative impact on semen parameters and male reproductive potential after recovery.

**Methods:**

In this longitudinal retrospective study, we enrolled 20 men who had COVID-19 disease. We compared sperm parameters in samples collected before COVID-19 and after infection (8.3 ± 4.8 months). We also evaluated the reproductive potential in pre- and post-COVID-19 infertility treatments of 8 self-controlled couples as well as in 40 cycles after COVID-19 infection of the male partner.

**Results:**

For most patients, we obtained results of more than one semen analysis before and after COVID-19. After adjusting for age, days of sexual abstinence, frequency of ejaculations and presence of fever, we found no significant difference over time in any semen parameter. The interval between COVID-19 infection and subsequent infertility treatments was 10.7 ± 7.5 months. There were no differences in the embryological and clinical outcomes of infertility treatments performed before and after male infection. One couple obtained a single pregnancy in the post COVID-19 IUI. Normal fertilization (65%), cleavage (99%) and blastocyst development (40%) rates in treatments performed after male infection were within the expected range of competencies. A total of 5 singleton and 1 twin clinical pregnancies were obtained, and 6 healthy children were born. A total of 10 blastocysts have been cryopreserved.

**Conclusion:**

Our data are reassuring that COVID-19 disease has no negative effect on semen quality and male reproductive potential when semen samples are collected three months or more after infection.

## Introduction

Severe acute respiratory syndrome coronavirus 2 (SARS-CoV-2) is a single-stranded RNA coronavirus and it causes Coronavirus disease 2019 (COVID-19). The SARS-CoV-2 consists of a viral envelope containing the RNA genome and a spike protein, which plays a key role during infection (1). Expression of both angiotensin-converting enzyme 2 (ACE2) and transmembrane protease serine 2 (TMPRSS2) on the surface of the host cell is required for viral entry: the spike protein binds to ACE2 and the TMPRSS2 activates cellular proteases to cleave the spike protein. The virus enters, its RNA is released, and genome replication and transcription begin (2).

The ability of SARS-CoV-2 to cross the blood-testis barrier and affect male fertility is still under discussion. The ACE2 is highly expressed in both Sertoli and Leydig cells and spermatogonia, as well as TMPRSS2A in the male genital tract, making the male reproductive system a potential target for SARS-CoV-2 (3–5). In addition, testosterone and androgen receptors, which are co-regulators of ACE2 and TMPRSS2 expression, probably help in the internalization of SARS-CoV-2 in the male reproductive organs (6). Moreover, a polymorphism of the androgen receptor may be responsible for the male predisposition to the most severe form of the disease. A consequent reduction in androgenic signaling exposes the host to an excessive inflammatory response, leading to the multi-organ damage typical of severe COVID-19 (7). Despite the expression of both ACE2 and TMPRSS2, the prostate and seminal vesicles have not been commonly described to be impacted in COVID-19. Otherwise, the testis resulted vulnerable to SARS-CoV-2, as there are reports of orchitis resulting in fibrosis and further loss of testicular function (8).

Except for Li et al. (9) who detected SARS-CoV-2 in semen of COVID-19 patients, subsequent studies did not find the virus in semen of men with active disease and those who recovered (10–17). Systematic reviews concluded that the likelihood of the virus being in the sperm of patients with COVID-19 is very low (18, 19).

Regardless of a local infection in the testis, COVID-19 disease may have temporary or permanent adverse effects on male fertility (20, 21). They may involve endocrine changes in luteinizing hormone and testosterone (22) and alterations in semen parameters (2, 9, 10, 15, 16, 22–25), reasonably due to fever or through a systemic inflammatory environment.

The above studies compared semen parameters of patients after SARS-CoV-2 infection with those of a similar demographic control group. As far as we know, only three studies have evaluated the effect of SARS-CoV-2 infection on semen parameters in a group of patients before and after coronavirus infection, reporting uneven data on the harmful consequences on sperm concentration, motility and morphology (26–28).

In addition, to date there is not enough data on the reproductive potential of men after COVID-19 disease, i.e., by evaluating the outcome of infertility treatments. Two published studies concluded that there is no evidence that a history of SARS-CoV-2 infection in men may adversely affect their fertility and performance in the subsequent assisted reproduction technique (ART) cycles, except for reduced embryo quality and decreased blastocyst formation rate and available blastocyst rate (28, 29). It should be noted that these data were collected only in 2 self-controlled couples who underwent ART treatments before and after COVID-19 infection of male partner (29) or by matching 50 couples with a history of previous SARS-CoV-2 infection in male partners with couples with similar baseline characteristics apart from their exposure to SARS-CoV-2 infection (28).

Prompted by the above limited information, we aimed to compare the seminal parameters of infertile male patients before and after SARS-CoV-2 infection, and to evaluate their reproductive potential in infertility treatments performed after their recovery.

## Materials and methods

### Study population and study design

This longitudinal retrospective study was carried out in a public tertiary level fertility center. We enrolled 20 non-azoospermic men undergoing fertility treatments at the Physiopathology of Human Reproduction, IRCCS Ospedale Policlinico San Martino, Genova, Italy, and who had COVID-19 disease, with a positive nasopharyngeal swab for SARS-CoV-2 infection. Semen parameters were compared before and after SARS-CoV-2 infection from February 2020 to December 2021. Four out of 20 patients had oligoastenospermia before contracting COVID-10 disease, 2 had oligospermia, and 2 had astenospermia. For each patient we also collected information about age, the degree of COVID-19 disease, the date of SARS-CoV-2 infection, and the second semen analysis.

**Figure 1:**
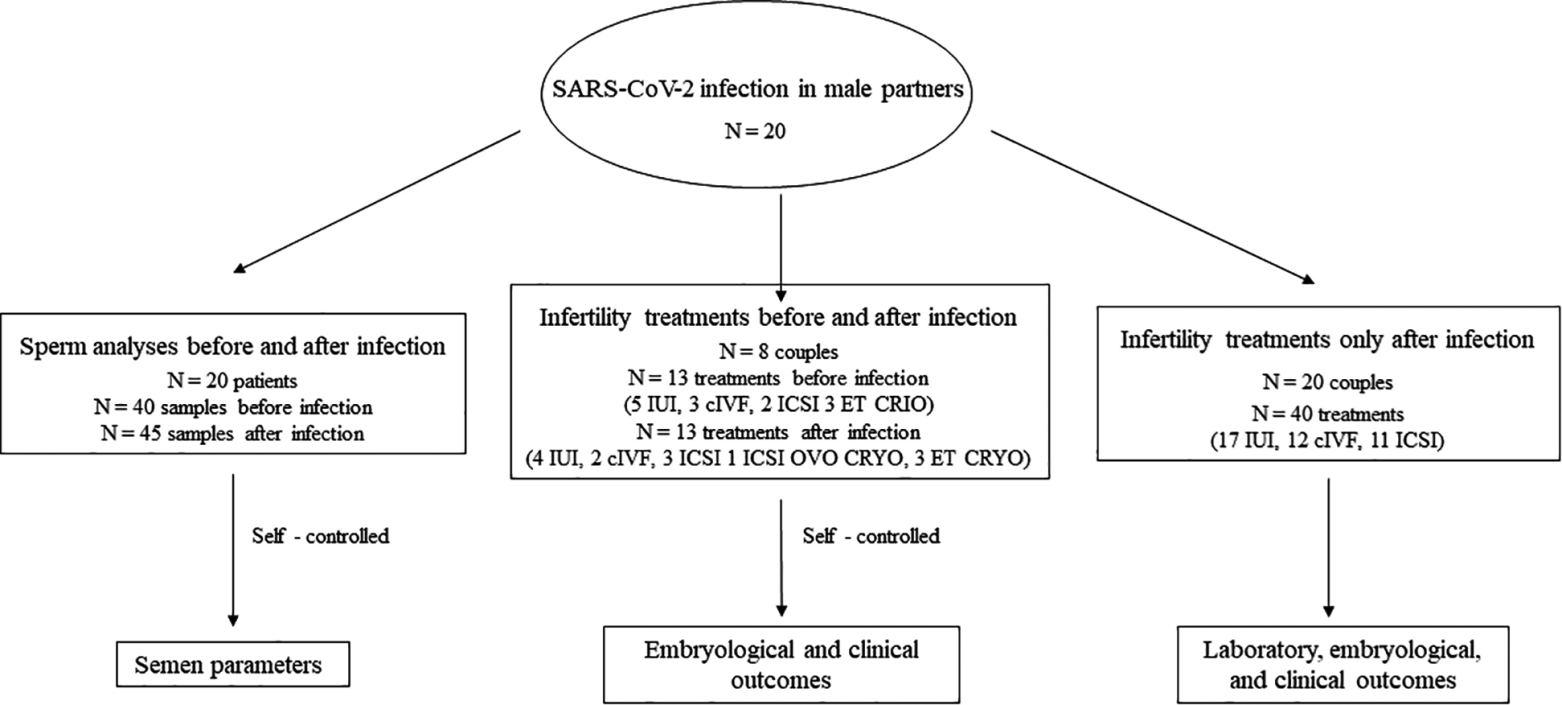
Study design.

As a secondary analysis, we evaluated the male reproductive potential in a subgroup of 8 men who performed intrauterine inseminations (IUI), ART cycles (conventional *in vitro* fertilization (cIVF), intracytoplasmic sperm injection (ICSI), and cycles from frozen embryos/oocytes) before and after COVID-19 infection. The reproductive potential of men was evaluated considering the embryological and clinical outcomes of the infertility treatments performed before and after COVID-19 infection, which were assessed and paired-matched for each couple. In addition, we included analysis of 40 infertility treatments (namely, 17 IUI, 12 cIVF, 11 ICSI) performed by all 20 couples after male infection. The study design is shown in Figure 1.

The study was approved by the Ethics Committee of Regione Liguria (approval n. 12609/2022).

### Semen analysis

Semen samples were collected by masturbation in a sterile container after 2–5 days of abstinence. The basic analysis, including semen volume, sperm concentration and motility, was performed following WHO guidelines (30, 31). For uniformity, the results of the semen parameters were reviewed (for “old” samples) and evaluated (for samples analyzed after July 2021) in accordance with WHO 2021 guidelines (31).

### Infertility treatments

Semen samples were prepared for infertility treatments using two-layer density gradient method (Sydney IVF Sperm Gradient, Cook Medical, Sydney, Australia). In ART cycles, controlled ovarian hyperstimulation, oocyte recovery, embryo culture, and morphological evaluation of embryos were performed as described previously (32). Oocytes were fertilized with cIVF or ICSI. Embryo transfer (ET) was generally performed 72 h after oocyte collection. However, based on of the number of embryos available, the ET could be performed on day 2 (if the patient had only one to two fertilized oocytes) or day 5 (if the patient had at least four good quality cleavage-stage embryos). Surplus blastocysts were cryopreserved.

The serum beta hCG test was performed 14 days after IUI or oocyte recovery. The presence of a gestational sac at the 6rd-7th gestational week was defined as a clinical pregnancy. Miscarriage was defined as a loss of pregnancy after ultrasonographic detection of a gestational sac.

### Statistical analysis

Descriptive statistics are reported as means ± standard deviation (SD) or median and interquartile ranges (IQRs) for continuous variables, and as absolute frequencies and percentages for categorical variables. To assess the impact of COVID-19 disease on primary endpoints (semen volume, sperm concentration and motility) we compared semen parameters in 20 patients who collected semen samples before and after coronavirus infection. For this purpose, we developed a linear mixed model to compare slope before and after COVID-19 disease. Dependence in data due to repeated measures was represented by a random intercept per individual and a self-regressive R covariance matrix was used as a correlation structure. The default REML estimation method was used for covariance parameters. The Kenward Roger method was used to compute the degrees-of-freedom for testing fixed effects. We adjusted the model using age, days of sexual abstinence, frequency of ejaculations and presence of fever as fixed effects.

Outcomes of infertility treatments performed before and after recovery from COVID-19 of male partner were paired-matched for each couple by paired student's *t*-test and Chi-square, as appropriate. Analyses were carried out by SAS version 9.4 (SAS Institute, Cary, NC) and MedCalc® software (Ostend, Belgium).

Birthweights of newborns were expressed as percentile and standard deviation score (SDS) for gestational age, according to the Italian reference curves (33).

## Results

### Semen parameters before and after COVID-19 disease

The characteristics of men enrolled in the study and the semen parameters of samples collected before and after COVID-19 disease are shown in Table 1. Fifteen patients had mild/moderate symptoms, 1 severe symptoms, and 4 were asymptomatic. Altogether, 11 patients (55%) had a fever for at least 2 days during the disease. All patients had their first negative nasopharyngeal swab after 10–15 days on their positive test, including the patient who had severe COVID-19 symptoms.

**Table 1:** Demographic and clinic characteristics of the men enrolled in the study and semen parameters before and after COVID-19 disease.

	Pre-COVID-19	Post-COVID-19
**All patients (*n* = 20)**		
Age, years	35.1 (33.7–36.6)	37.5 (36.5–45.6)
Sexual abstinence, days	4.0 (3.0–4.0)	3.0 (3.0–4.0)
Volume, ml	2.8 (2.3–3.2)	2.7 (2.2–3.1)
Sperm concentration, million/ml	24.5 (18.3–33.3)	32.0 (22–53.6)
Total motility, %	45.0 (40.0–63.0)	50.0 (35.0–55.0)
Progressive motility, %	36.0 (35.0–47.8)	35.0 (30.0–40.0)
**Patients who had not fever (*n* = 9)**		
Age, years	35.8 (33.5–39.7)	39.9 (36.7–46.1)
Sexual abstinence, days	4.0 (3.0–4.2)	3.0 (3.0–4.0)
Volume, ml	2.9 (2.3–3.5)	2.5 (2.2–3.4)
Sperm concentration, million/ml	22.0 (9.6–35.1)	42.0 (22.0–66.1)
Total motility, %	45.0 (40.0–56.0)	48.0 (40.0–55.0)
Progressive motility, %	38.5 (34.2–47.5)	40.0 (35.2–45.0)
**Patients who had fever (*n* = 11)**		
Age, years	35.1 (33.8–36.4)	37.4 (36.2–38.5)
Sexual abstinence, days	4.0 (3.0–4.0)	3.0 (3.0–4.0)
Volume, ml	2.6 (2.0–3.5)	2.8 (2.0–3.9)
Sperm concentration, million/ml	26.0 (18.5–35.8)	31.0 (16.7–63.5)
Total motility, %	45.0 (35.0–65.0)	35.0 (28.8–55.0)
Progressive motility, %	36.0 (30.3–51.7)	30.0 (20.7–38.8)

Values are median. In the brackets the interquartile range is reported.

At the time of disease, no patient was vaccinated. Fifteen patients received vaccination later and we collected the date of their vaccination in 10 of these patients. Of these, 14 semen samples were collected after vaccination, representing 31% (14/45) of the total post-COVID semen samples analyzed. We have not considered this issue as a potential bias because we have shown that semen parameters after COVID-19 vaccination did not reflect any detrimental effect of vaccination (34).

Pre-COVID-19 samples were obtained after a median abstinence period of 4 days (IQR 3.0–4.5) and post-COVID-19 samples after a median of 3 days (IQR 2.5–4.0), without any significant difference. Post-COVID-19 samples were obtained after an average of 8.3 ± 4.8 months from the positive PCR test for SARS-CoV-2 and at least 3 months after negativization of patients. For most patients, we obtained results of more than one semen analysis before (mean number: 2.0 ± 1.2) and after (mean number: 3.1 ± 1.4) COVID-19.

After adjusting for age, days of sexual abstinence, frequency of ejaculations and presence of fever, we found no significant difference between groups in the rate of change over time in any of the semen outcome (Table 2). In fact, the difference between the slopes before and after COVID-19 disease were not statistically significant in any of the semen parameters.

**Table 2:** Estimates and associated 95% confidence intervals (CI) for the slope β. The models are adjusted for age, days of sexual abstinence, frequency of ejaculations and presence of fever. .

	Slope (*β*) pre-COVID-19	*p*-value	Slope (β) post-COVID-19	*p*-value	Difference in slopes	*p*-value
Volume, ml	−0.05 (−0.23–0.12)	0.535	−0.03 (−0.11–0.06)	0.557	0.03 (−0.15–0.21)	0.756
Sperm concentration, million/ml	2.75 (−1.92–7.43)	0.240	2.34 (−0.09–4.77)	0.058	−0.41 (−5.27–4.46)	0.865
Total sperm count	9.55 (−4.95–24.06)	0.190	7.79 (0.21–15.37)	0.044	−1.76 (−16.84–13.32)	0.813
Progressive motility, %	1.56 (−0.29–3.41)	0.096	0.91 (−0.03–1.85)	0.058	−0.65 (−2.58–1.28)	0.497
Total motility, %	2.55 (0.65–4.45)	0.010	1.07 (0.06–2.09)	0.039	−1.47 (−3.45–0.50)	0.138
TMSC	5.58 (−3.76–14.93)	0.234	5.63 (1.29–9.97)	0.012	0.04 (−9.85–9.94)	0.993

Values are median. In the brackets the interquartile range is reported. TMSC: total motile sperm count.

Six patients were oligospermic before SARS-CoV-2 infection (mean concentration 8.7 ± 4.2 million/ml). Among them, only one had a fever during COVID-19 and his sperm concentration decreased from 11 to 3.5 million/ml after infection. All other 5 patients were normozoospermic after the disease (median concentration: 30.0 million/ml, IQR 21.0–68.0).

### Infertility treatments performed before and after recovery from COVID-19 of male partner

Eight couples who performed infertility treatments at our center before the COVID-19 pandemic resumed infertility treatments after recovery from COVID-19 of the male partner and a total of 13 treatments were performed, namely, 4 IUI, 2 conventional IVF (cIVF), 4 ICSI including one from frozen oocytes and 3 cycles from frozen blastocysts. The interval between the COVID-19 infection (positive polymerase chain reaction test) and subsequent infertility treatments was an average of 10.7 ± 7.5 months. Clinical characteristics of patients and details of their attempts to treat infertility, before and after the COVID-10 infection, are shown in Table 3. There were no differences in laboratory, embryological and clinical outcomes of fertility treatments performed before and after male infection. One couple obtained a single pregnancy in the post COVID-19 IUI. After COVID-19, five gestations after an ART cycle (of which one from frozen oocytes) ended in successful healthy newborns.

**Table 3:** Embryological, laboratory and clinical outcomes of the fertility treatments performed before and after recovering from COVID-19 disease of the male partner.

	Pre-COVID	Post-COVID	*p*-value
**Paired IUI**			
Number of couples	3	3	–
Number of IUI cycles	5	4	–
Pre-capacitation TMSC (millions, mean ± SD)	52.6 ± 17.7	86.4 ± 89.6	0.634*
Clinical pregnancy per couple, *n* (%)	2/3 (67)	1/3 (33)	0.987
Miscarriage rate, *n* (%)	0/3	0/3	–
**Paired ART (cIVF or ICSI) cycles**			
Number of couples/fresh cycles	5	5	–
Female age, years, mean ± SD	33.4 ± 4.8	33.8 ± 5.9	0.625
Male age, years, mean ± SD	35.8 ± 1.8	36.9 ± 1.7	0.573
Fertilization rate, %, mean ± SD	63 ± 33	51 ± 38	0.625
Cleavage rate, %, mean ± SD	100 ± 0	100 ± 0	–
Top quality embryos, %, mean ± SD	57 ± 23	49 ± 36	0.625
Blastocyst formation rate, *n* (%)	5/16 (31)	8/21 (38)	0.925
Pre-capacitation TMSC, millions, mean ± SD	34.8 ± 24.5	61.7 ± 23.9	0.180*
Implantation rate, *n* (%)	0/5	2/5 (40)	0.429
Pregnancy rate per cycle, *n* (%)	0/5	2/5 (40)	0.429
Pregnancy rate from frozen oocytes/embryos per cycle, *n* (%)	2/3 (67)	1/4 (25)	0.734
Cumulative pregnancy rate per couple, *n* (%)	2/8 (25)	3/9 (33)	0.863
Miscarriage rate, *n* (%)	2/2 (100)	0/3	0.192

Values are mean ± standard deviation (SD) unless otherwise stated. TMSC: total motile sperm count.

* *P*aired samples *t*-test.

The 20 couples performed a total of 40 infertility treatments [17 IUI and 23 ART (12 cIVF, 11 ICSI) cycles] after recovery from COVID-19 disease of the male partner. The interval between COVID-19 infection and subsequent infertility treatments was an average of 10.5 ± 5.2 months. Normal fertilization (65%), cleavage (99%) and blastocyst development (40%) rates were within the expected range of competences according to the Vienna criteria (35). A total of 5 singleton and 1 twin clinical pregnancies were obtained. Out of the six patients who successfully conceived after COVID-19 disease, 3 were normozoospermic, 2 astenospermic, and one oligospermic at the time of the cycle. A total of 10 blastocysts have been cryopreserved in 4 cycles, thus further embryo transfers will be performed.

As for the pregnancy outcomes, the twin pregnancy was spontaneously reduced to singleton and a total of 6 healthy children were born from the post-COVID cycles. The perinatal characteristics of children are detailed in Table 4. No stillbirths as well as no malformations were recorded among the newborns.

**Table 4:** Neonatal characteristics of live births in post COVID-19 cycles.

Parameter	
N. live births	6
N. lost follow-up	1
N. ongoing pregnancies	0
N. singletons	6
N. twins	0
Birthweight (grams)	2986 ± 1236
N. birthweight <2,500 g	1
Gestational age (weeks)	38.0 ± 2.4
N. prematurity <37 weeks	2
Birthweight centiles	43.5 ± 26.1
SDS-score	−0.2 ± 0.9

Values are mean ± standard deviation (SD) unless otherwise stated.

## Discussion

In this study we found that semen parameters did not reflect any detrimental effect of COVID-19 disease. In addition, we have not observed any influence of COVID-19 infection on the performance of male patients in their subsequent infertility treatments.

SARS-CoV-2 enters host cells through ACE2 receptors and testes are characterized by expression of ACE2 and TMPRSS2 (2), making the seminal fluid susceptible to infection. Moreover, the impact of COVID-19 on male reproductive health may be worsened in men due to genetic mechanisms. In fact, a polymorphism of the androgen receptor may be responsible for the male predisposition to the most severe form of the disease. A subsequent reduction in androgenic signaling exposes the host to an excessive inflammatory response, leading downstream to the multi-organ damage typical of severe COVID-19 (7). In addition to men generally having more comorbid conditions than women, elevated androgen levels have been identified as a potential mechanism for viral cell entry: the ability of the androgen receptor to upregulate ACE2 and TMPRSS2 gene may be a reason for the worst disease severity (8).

Several studies investigated SARS-CoV-2 in semen of recovered and acutely infected patients (9–12, 14, 25). With the exception of one study that found SARS-CoV-2 RNA in semen samples (9), all other studies did not detect viral RNA in semen of patients with active or infection that is resolving (10–17). The exact impact of SARS-CoV-2 infection on seminal parameters is still under discussion. In this study we did not find significant difference in the rate of change over time in any of the semen analyzed after COVID-19 infection. These results are consistent with those previously reported (28, 35, 36). However, several studies reported changes in semen parameters after recovery from the disease (9, 16). The severity of COVID-19 may be one of the possible reasons for this discrepancy, as well as the selection of controls, that is, the absence of paired analyses with semen parameters before infection (9, 16, 23–25). In this regard, we performed a self-controlled study to eliminate individual differences. Another critical issue is the time between infection and semen analysis. A deteriorating impact of COVID-19 on semen parameters has been demonstrated immediately after infection (23, 24), while semen analysis in our study was performed at least 3 months after viral infection, so a period passed for the recovery of the reproductive function of the participants. This may be the main reason for the inconsistency between the results, and confirm that male reproductive function may be impaired in a short period of time, while in the long term it may return to normal (15, 27, 28). In addition, recent data remarked that COVID-19 does not appear to cause direct damage to testicular function, while indirect damage appears to be transient (37, 38). Overall this can lead to the conclusion that temporary variations in semen quality can be attributed to acute febrile disease, since hyperthermia is known to have this effect. However, in our study, the presence of fever during COVID-19 disease was not correlated with sperm quality parameters, indicating that there are many indirect ways through which the pituitary-gonadal axis and spermatogenesis may be disrupted, as immunological factors and/or disruption of the blood-testis barrier (15), cytokine imbalance, and drugs (38).

As for the effect of COVID-19 on male reproductive potential, it is still a little studied topic. Whereas the male genome begins to activate after fertilization and occurs at the blastocyst stage (39), the results of ART cycles are a more accurate indicator of sperm reproductive competence than semen analysis. Contrary to what was previously reported by Wang et al. (28), we did not observe any negative impact on the development competence of embryos in the treatments of 8 self-controlled couples as well as in 23 ART cycles performed after COVID-19 infection of the male partner. It is noteworthy that in most of our couples at least five months had elapsed between the diagnosis of SARS-CoV-2 infection and the ART cycle.

Overall our findings suggest that men of reproductive age seeking to conceive should be warned that the quality of sperm after COVID-19 infection could be suboptimal and advised to postpone infertility treatments for at least 3 months (duration of spermatogenesis), with the aim of recruiting healthy spermatozoa that have not been exposed to the disease during their development. More extensive studies with a longer follow-up will be needed to confirm this and to determine whether permanent harmful effects may occur in a minority of men (15). In addition, if SARS-CoV-2 infection in males adversely impacts embryonic epigenetics, resulting in health disorders of newborns and offspring, follow-up of children should be recommended.

To the best of our knowledge, the current study is the largest to enroll couples undergoing infertility treatments with a history of SARS-CoV-2 infection of the male partner. Our study has strengths and limitations. The main strength is that this study provides information on COVID-19 effects on semen parameters in a group of patients who collected semen samples before and after coronavirus infection with a follow-up time of up to twelve months. Similarly, we evaluated the embryological, laboratory and clinical outcomes of fertility treatments performed in couples before and after recovering from COVID-19 disease of the male partner. Moreover, most patients have had more than one semen analysis both before and after infection, making comparisons of our results accurate despite the well-known dynamic nature of sperm characteristics.

However, our study is not without limitations. First, it was a single-center longitudinal retrospective study with a small sample size. Secondly, the lack of long-term follow-up as well as data on live childbirth and further cumulative pregnancy outcomes. Third, most of our participants had an infection with only mild clinical symptoms, which may not account for the exact impact of SARS-CoV-2 on fertility treatment results. Fourthly, there is a limited generalization because no data on sperm quality have been collected in fertile patients.

Our data are reassuring that COVID-19 disease has no negative effect on semen quality and male reproductive potential when semen samples are collected three months or more after infection.

## Data Availability

The raw data supporting the conclusions of this article will be made available by the authors, without undue reservation.
